# From dyad to triad: a survey on fathers’ knowledge and attitudes toward breastfeeding

**DOI:** 10.1007/s00431-021-04034-x

**Published:** 2021-03-29

**Authors:** Beatrice Letizia Crippa, Alessandra Consales, Daniela Morniroli, Flavia Lunetto, Maria Enrica Bettinelli, Patrizio Sannino, Serena Rampini, Lidia Zanotta, Paola Marchisio, Laura Plevani, Maria Lorella Giannì, Fabio Mosca, Lorenzo Colombo

**Affiliations:** 1 Fondazione IRCCS Cà Granda Ospedale Maggiore Policlinico, NICU, Milan, Italy; 2 Department of Clinical Sciences and Community Health, Università degli Studi di Milano, 20122 Milan, Italy; 3 Direzione Professioni Sanitarie, Fondazione IRCCS Ca’ Granda Ospedale Maggiore Policlinico, Milan, Italy; 4 Fondazione IRCCS Ca’ Granda Ospedale Maggiore Policlinico, 20122 Milan, Italy; 5 Università degli Studi di Milano, 20122 Milan, Italy

**Keywords:** Fathers, Paternal involvement, Knowledge, Attitude, Breastfeeding

## Abstract

Fathers are known to impact breastfeeding outcomes. We aimed to explore paternal knowledge and attitude toward breastfeeding, and possible association with breastfeeding rates at discharge. In this cross-sectional study, we enrolled 200 fathers of healthy term neonates. At discharge, fathers were asked to rate their degree of agreement to 12 items on a 5-point Likert scale. A total score was obtained from their answers. Univariate binary logistic regression analysis was used to verify if the total score was predictive of exclusive breastfeeding at discharge. A multivariable logistic regression model was then used to adjust for possible confounders. ROC analysis was performed, and a Youden’s total score cut-off value was determined to define total score’s performance in predicting exclusive breastfeeding at discharge. Fathers showed a solid knowledge of maternal (87%) and neonatal (98%) benefits of breastfeeding, skin-to-skin (99.5%), rooming-in (79%), and responsive feeding (67.5%); conversely, only 51% knew about the recommended use of pacifiers. Fathers felt personally involved in babies’ feeding in 79% of cases. An association was found between total score and exclusive breastfeeding at discharge at univariate (OR: 1.07, *p* = 0.04) but not at multivariable analysis (OR: 1.07, *p* = 0.067). ROC analysis was not statistically significant (AUC 0.58, *p* = 0.083).

*Conclusion*: By using a novel instrument aimed at quantifying fathers’ knowledge and overall attitude toward breastfeeding, this study underlines the importance of including fathers in the promotion of breastfeeding. Expanding the classic mother-baby dyad to a more modern mother-father-baby triad may impact breastfeeding outcomes at discharge.

**Table:** 

**What is known:** *• Social support plays a major role in improving breastfeeding outcomes*. *• Fathers may greatly influence initiation and duration of breastfeeding; the more they know, the more helpful they can be*. **What is new:** *• A multidisciplinary team created a structured questionnaire aimed at quantifying fathers’ knowledge and attitude toward breastfeeding*. *• The association between a higher questionnaire total score and exclusive breastfeeding rates at discharge highlights the importance of including fathers in the promotion of breastfeeding, as part of the breastfeeding team*.

**Supplementary Information:**

The online version contains supplementary material available at 10.1007/s00431-021-04034-x.

## Background

Breastfeeding is the cornerstone of newborns’ nutrition. The World Health Organization (WHO) and the United Nations Children’s Fund (UNICEF) recommend it as the exclusive mode of feeding of the infant for its first 6 months of life [1], given its well-known short- and long-term benefits for mother, child, and society [2–4]. The Baby-Friendly Hospital Initiative (BFHI) has been stressing healthcare professionals’ role in the protection and promotion of breastfeeding since 1991 [5]. Nevertheless, outside of hospital facilities, social support plays an important role in determining breastfeeding outcomes [6]. Indeed, the Ten Steps to Successful Breastfeeding [7], which the BFHI relies upon, advise to facilitate families at discharge with timely access to ongoing support and care (10^th^ Step), thus ensuring continuity of care. Along this line, many countries, including Italy, have implemented the Baby-Friendly Community Initiative (BFCI), which is considered as an expansion and integration of the BFHI [8–10]. The global standards for the BFCI require the implementation of 7 Steps [9]. Among these steps, the third one advises to extend breastfeeding education to the whole family, and the sixth one encourages to provide a welcoming atmosphere for breastfeeding families.

At the same time, the evolution the role of fathers has undergone over the centuries, from a patriarchal bread-winning ideal to the modern involved co-parent [11], has facilitated the transition to a more family-centered approach of care.

A change of perspective has therefore been advocated to promote and facilitate the involvement of fathers in their newborns’ health, thus expanding the center of attention from the classic mother-baby dyad to the more complex mother-father-baby triad [12].

Fathers’ attitudes have a significant impact on mothers’ breastfeeding decisions [13]. Fathers’ psychological and practical support influences initiation and duration of breastfeeding [14], at the same time acting as a confidence booster for mothers, who develop a higher self-efficacy if they feel supported by their partners [15]. Supportive actions are heterogeneous in nature [16], but what drives them is the awareness of the importance of breastfeeding [17]. The more a father knows about breastfeeding benefits and management, the more likely he is to influence its initiation and continuation [13]. Moreover, according to a recent meta-analysis, targeting fathers in breastfeeding promotion in prenatal and postnatal settings improves exclusive breastfeeding rates at 4 and 6 months [18]. Therefore, it has been advocated that healthcare professionals favor a more meaningful engagement of fathers in their newborns’ well-being, especially focusing on their key role of breastfeeding support [19].

However, little is known about what fathers in Italy know and how they feel about breastfeeding and its determinants and facilitators.

The present study aimed to investigate paternal knowledge and attitude toward breastfeeding, and their association with breastfeeding rates at discharge, in a cohort of fathers from an Italian neonatal tertiary referral center. Moreover, we created a novel instrument aimed at quantifying fathers’ knowledge and overall attitude toward breastfeeding, and assessed its performance in predicting exclusive breastfeeding at discharge.

## Methods

### Design and setting

A cross-sectional study was conducted in May 2019 in the postnatal unit of our hospital, a tertiary referral center for neonatal care, which operates in compliance with the BFHI principles. Indeed, our hospital promotes and supports breastfeeding in all mother-infant dyads throughout hospital stay, following the principles of the BFHI. We have a written breastfeeding protocol, and we can rely on the presence of an International Board Certified Lactation Consultant (IBCLC) in situ. Nurses are actively involved in the promotion of breastfeeding and support at the bedside. At birth, skin-to-skin practice is explained to all new parents and facilitated by pulse oximetry monitoring in case of skewed healthcare professional:dyad ratio. Likewise, rooming-in is encouraged and safety practices explained to both parents to ensure the well-being of the newborn. Our policies regarding rooming-in are described in greater detail elsewhere [20].

The institutional Ethics Committee approved the present study. Both mothers and fathers provided written informed consent for both the questionnaire and access to neonatal and maternal medical charts.

### Sample

We enrolled a convenience sample of fathers of healthy term neonates born at our hospital after an uneventful single pregnancy in May 2019. We excluded (i) fathers without a good oral and written comprehension of the Italian language, (ii) fathers of neonates hospitalized in the Neonatal Intensive Care Unit (NICU) and/or affected by any condition that could interfere with breastfeeding, (iii) fathers of neonates small for gestational age (< 10° percentile), (iiii) fathers of twins, and (iv) fathers of neonates whose mothers had contraindications to breastfeeding (i.e., previous breast surgery, drugs incompatible with breastfeeding, HIV, or human T cell lymphotropic virus infection) and/or had chosen not to breastfeed.

Fathers of twins and neonates small for gestational age were excluded to obtain a homogeneous sample, considering the breastfeeding difficulties and lower breastfeeding rates reported in these newborns [21, 22].

Partners of mothers with contraindications to breastfeeding were excluded based on the assumption that their attitude toward breastfeeding might be somewhat biased by the psychological impact of the impossibility to breastfeed.

### Data collection and procedures

At discharge, a dedicated neonatologist proposed to fathers a self-administered structured questionnaire. The questionnaire took approximately 10 min to be filled out and was collected by the same healthcare professional 20 min after being handed out.

Obstetric charts and infants’ computerized medical charts (Neocare, i&t Informatica e Tecnologia Srl, Italy) were used to collect the basic characteristics of mothers and fathers (i.e., age, ethnicity, level of education, marital status, parity), current mode of feeding, and previous feeding experiences at discharge (none, exclusive breastfeeding, mixed feeding, bottle feeding).

For reporting purposes, maternal and paternal level of education was expressed in terms of years of education: ≤ 13 (primary school, secondary school, and/or high school diploma) and > 13 years (university degree).

Mode of feeding was defined according to the WHO definitions [1].

All other data were obtained from the questionnaire.

### Instrument

The structured self-administered questionnaire used for the purposes of the present study was created by a multidisciplinary team consisting of a neonatologist, an obstetrician, an IBCLC, and a pediatric nurse, inspired by the tool used by Brown et al. [23]. All the members of the multidisciplinary team work for our institution, a BFHI-compliant facility. The questionnaire was structured to follow the WHO/UNICEF Ten Steps for Successful Breastfeeding [7] and it was developed through a series of meetings during which each member of the multidisciplinary team was asked to express their agreement (or lack thereof) on the inclusion of the various items. Based on the percentage of team members agreeing with the inclusion of each item in the questionnaire, the items were included (≥ 50%) or rejected (< 50%). The newly created questionnaire (Supplementary Table 1) was administered to a sample of 50 fathers (40 Italians and 10 foreigners) to ascertain items’ comprehension; these 50 fathers were not considered part of the present study population, nor were they included in the statistical analysis. Fathers were asked to express any concern regarding the questionnaire in a specific section of the document provided. No specific issues emerged. No item of the questionnaire was subsequently changed.

The questionnaire showed acceptable internal consistency (Cronbach’s alpha = 0.7).

The questionnaire encompassed 12 items divided into 9 sections (Supplementary Table 1). Fathers were required to rate their degree of agreement to each item on a 5-point Likert scale, ranging from “Strongly Disagree” (= 1 point) to “Strongly Agree” (= 5 points). A total score (min. 12, max. 60 points) for each father was obtained by adding up the points assigned to the various items. The first seven sections addressed 8 out of the 10 Steps for Successful Breastfeeding [7], as explained below: Section 1: antenatal care (3^rd^ Step), Section 2: perinatal care (4^th^ Step), Section 3: breastfeeding support (5^th^ Step), Section 4: rooming-in (7^th^ Step), Section 5: responsive feeding (8^th^ Step), Section 6: use of pacifier (9^th^ Step), Section 7: staff competency, and information received at discharge (2^nd^ and 10^th^ Steps). Sections 8 and 9 investigated fathers’ opinions on breastfeeding impact on everyday life and breastfeeding in public.

In order not to compromise the authenticity of the responses, fathers were asked to fill out the questionnaire independently, without sharing their answers with their partner.

### Statistical analyses

Categorical variables were expressed as frequencies. Continuous variables were expressed as mean ± standard deviation (SD) or median [inter-quartile range]. Non-parametric tests were used to assess differences in total scores between subgroups. The variables considered were basic characteristics of fathers (age, ethnicity, level of education) and breastfeeding experience (maternal parity and previous feeding experiences at discharge). The total score was obtained by adding up the points assigned to each item in the questionnaire (min 12, max 60 points): a higher score was considered as indicative of greater knowledge and positive attitude toward breastfeeding.

**Fig. 1:**
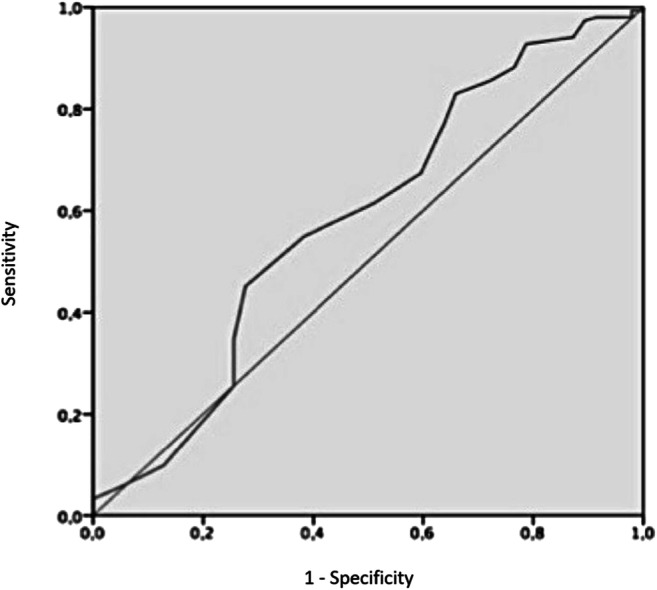
Receiver operating characteristic (ROC) curve for total score values obtained from all participants. AUC 0.58, *p* = 0.083, 95% CI 0.485–0.683

Univariate binary logistic regression analysis was used to verify if the total score was a predictor of exclusive breastfeeding at discharge. Likewise, univariate analysis was used for variables reported in the literature to be predictive of exclusive breastfeeding [24, 25]. Variables found to be significant at univariate analysis were entered into a multivariable logistic regression model to adjust for possible confounders. ROC analysis (Fig. 1) was then performed, and a Youden’s total score cut-off value was determined to define the total score’s performance in predicting exclusive breastfeeding at discharge.

Statistical analyses were performed using SPSS version 25 Statistic Software Package (SPSS Inc., Chicago, IL, USA).

For reporting purposes, data from the questionnaire are shown categorized into three groups: agree (Likert scale 4 and 5), disagree (Likert scale 1 and 2), and neutral (Likert scale 3).

## Results

The total eligible population consisted of 210 fathers. Seven of them (3.3%) were not included in the study based on the exclusion criteria, and 3 (1.4%) refused to participate. The enrolled population included 200 fathers who completed the questionnaire. Basic characteristics of the mother-father couples enrolled are summarized in Table 1.

**Table 1:** Basic characteristics of study population

**Sociodemographic features**		
	**Fathers (*n*** **= 200)**	**Mothers (*n*** **= 200)**
Age, years (mean ± SD)	37.2 ± 5	34.6 ± 5
Ethnicity, *N* (%)		
Italian	191 (95.5)	175 (87.5)
European	3 (1.5)	15 (7.5)
Other	6 (3)	10 (5)
Level of education, *N* (%)		
≤ 13 years	86 (43)	67 (33.5)
> 13 years	114 (57)	133 (66.5)
Marital status, *N* (%)		
Married	120 (60)	
Unmarried relationship	80 (40)	
**Delivery and breastfeeding experience**		
	**Mothers (*n*** **= 200)**	
Parity, *N* (%)		
Primiparous	136 (68)	
Multiparous	64 (32)	
Type of delivery, *N* (%)		
Spontaneous	112 (56)	
Cesarean section	88 (44)	
Previous feeding experience at discharge, *N* (%)		
None	139 (69.5)	
Exclusive breastfeeding	36 (18)	
Mixed feeding	15 (7.5)	
Bottle feeding	10 (5)	
Feeding at discharge, *N* (%)		
Exclusive breastfeeding	153 (76.5)	
Mixed feeding	35 (17.5)	
Bottle feeding	12 (6)	

This table presents the basic characteristics of the mother-father couples who participated in the study

The mean paternal and maternal age were 37.2 ± 5 and 34.6 ± 5 years, respectively. The sample comprised mainly Italian parents with a high level of education (> 13 years). More than half of the participants were married (60%).

Most mothers were primiparous (68%) and had a spontaneous delivery (56%). Only 36 mothers (18%) had previous experience of exclusive breastfeeding at discharge, while 139 (69.5%) had no experience at all. Exclusive breastfeeding rate at discharge was 76.5%.

The answers to the items assessed in the questionnaire are shown in Table 2. No missed data were reported. Most fathers were aware of breastfeeding benefits for infants (98%), mothers (87%), and society (64.5%), and 135 (67.5%) stated that they had received sufficient information on breastfeeding management during pregnancy. Almost all fathers believed in the importance of skin-to-skin contact after birth (99.5%) and rooming-in (79%). Most of them (79%) felt directly involved in breastfeeding their baby regardless of type of feeding (exclusive vs. non-exclusive breastfeeding, *p* = 0.752) and considered breastfeeding on demand beneficial (67.5%). Only 51% of fathers were aware of the recommended restrictions on pacifier use in the first month of life. Information received during hospital stay was considered clear by 87% of fathers. One-hundred and twenty-nine fathers (64.5%) thought that breastfeeding could lead to difficulties in everyday life, and 186 (93%) were supportive of breastfeeding in public.

**Table 2:** Answers to the self-administered questionnaire

Questions		Fathers (*n* = 200)		
		Agree	Disagree	Neutral
		*N* (%)	*N* (%)	*N* (%)
1	Antenatal care (3rd step)			
	I received sufficient information on breastfeeding management	135 (67.5)	65 (32.5)	-
	Breastfeeding offers health benefits for infants	196 (98)	2 (1)	2 (1)
	Breastfeeding offers health benefits for mothers	174 (87)	24 (12)	2 (1)
	Breastfeeding benefits society	129 (64.5)	59 (29.5)	12 (6)
2	Perinatal care (4th step)			
	Skin-to-skin contact after birth is a valuable opportunity	199 (99.5)	1 (0.5)	-
3	Breastfeeding support (5th step)			
	I feel personally involved in feeding my baby	158 (79)	42 (21)	-
4	Rooming-in (7th step)			
	Rooming-in affects breastfeeding initiation	158 (79)	30 (15)	12 (6)
5	Responsive feeding (8th step)			
	Breastfeeding on-demand is beneficial	135 (67.5)	54 (27)	11 (5.5)
6	Use of pacifier (9th step)			
	Breastfed infants should not use pacifiers in the first month of life	102 (51)	79 (39.5)	19 (9.5)
7	Staff competency and discharge (2nd and 10th step)			
	Information received during hospital stay and at discharge was clear	174 (87)	18 (9)	8 (4)
8	Breastfeeding does not complicate everyday life	59 (29.5)	129 (64.5)	12 (6)
9	Mothers can breastfeed wherever they are	186 (93)	14 (7)	**-**

This table provides details of the answers to the various items of the questionnaire given by fathers enrolled in the study

No difference in total score median values was found between the subgroups analyzed, based on the following variables: paternal age, level of education, ethnicity, maternal parity, and previous feeding experience at discharge (Supplementary Table 2).

The total score was found to be associated with exclusive breastfeeding at discharge at univariate analysis (OR: 1.07, *p* = 0.04; 95% CI 1.002–1.152). In a multivariable model including maternal age, parity, mode of delivery, and total score, parity and mode of delivery were found to be independently associated with exclusive breastfeeding at discharge (OR: 4.42 and 3.07, *p* = 0.0001 and 0.004, respectively), while total score was not (OR: 1.07, *p* = 0.067). When a total score of 50 was chosen as a cut-off value, it resulted in a sensitivity of 54.9% and a specificity of 61.7% in predicting exclusive breastfeeding at discharge. However, ROC analysis performed to assess the predictive power of the total score was not statistically significant (AUC 0.58, *p* = 0.083, 95% CI 0.485–0.683).

## Discussion

In the present study, fathers enrolled were reasonably well informed about breastfeeding. Almost all fathers were aware of the beneficial effects of breastfeeding on infants’ and mothers’ health, skin-to-skin contact, rooming-in practice, and responsive feeding. However, only half of them were aware of the recommendations on the use of pacifiers for breastfed infants. Moreover, fathers showed an overall positive attitude toward breastfeeding, although pointing out that breastfeeding does complicate everyday life, and generally felt personally involved in their babies’ feeding, regardless of type of feeding. An association was found between the total score of the questionnaire proposed to fathers and exclusive breastfeeding at discharge at univariate, although not at multivariable analysis.

It has been described how fathers play an important role in the initiation and duration of breastfeeding. According to Bar-Yam and Darby [26], fathers may influence four different aspects: the breastfeeding decision, assistance at first feeding, duration of breastfeeding, and risk factors for artificial feeding. Two systematic reviews [18, 27] reported how an increased paternal breastfeeding knowledge can positively affect breastfeeding outcomes (initiation, exclusivity, and continuation). Two randomized controlled trials showed how educating fathers for the role of “breastfeeding coach” has positive effects on breastfeeding in terms of increased initiation rate, reduced worry about low milk supply, and reduced premature breastfeeding cessation [13, 28]. Moreover, in a recent review, Sihota et al. highlighted the need for comprehensive antenatal support and education tailored for fathers of breastfed infants [29]. Interestingly, some authors have reported how fathers themselves want to know more about breastfeeding [13, 29]. Most fathers enrolled stated that they had received sufficient information either before their baby’s birth or during hospital stay or at discharge. The high percentage of answers in line with the BFHI principles demonstrates a generally solid knowledge of the subject.

A recent study by Chen et al. reported lower Quality of Life scores in fathers of breastfed infants than in fathers of bottle-fed infants, mainly due to the perceived more limited bonding opportunities with the baby [30]. Paternal postpartum depression is a worrying reality, connected with feelings of inadequacy and reduced self-efficacy often prompted by a sense of uselessness when compared to the mother’s nursing role [31]. Greater paternal involvement in breastfeeding may provide fathers with more occasions to bond with their newborn, thus proving beneficial for their mental health as well [31]. The fact that fathers in our study felt generally involved in their babies’ feeding, regardless of type of feeding, should therefore be regarded as a positive, well-boding result.

A high percentage of fathers interviewed declared to be in favor of breastfeeding in public, a possible sign of the changing times. Breastfeeding in public is still a controversial issue [32]: several studies have reported how it is often perceived by men as uncomfortable, embarrassing, and even distasteful [23, 33–35], showing a correlation with socio-economic status [34, 35] and cultural background [29, 36]. A significant push toward the rethinking of breastfeeding in public has been given by the implementation of the BFCI [9]. In particular, the 6^th^ Step of the BFCI [9] aims at the creation of breastfeeding-friendly environments, where nursing mothers can feel welcome. As Boyer pointed out in a recent paper, acceptance of breastfeeding in public is, first of all, a cultural issue that the government could help address by implementing programs that challenge current social norms [37].

Finally, at univariate analysis, an association was found between the total score obtained from the questionnaire and exclusive breastfeeding at discharge. Exclusive breastfeeding rate at discharge (76.5%) was comparable to what was previously reported in our population (75%) [38]; it resulted higher than the national (57.2%) and regional average (67.3%) [39, 40], and in line with the WHO/UNICEF Global Strategy for Infant and Young Child Feeding recommended rate of 75% [41]. The higher the score is (hence paternal knowledge of and positive attitude toward breastfeeding), the higher the probability of exclusive breastfeeding at discharge is. The association between a higher questionnaire total score and exclusive breastfeeding rates at discharge may indicate a potential positive influence of fathers on newborn’s feeding choices at discharge. However, multivariable analysis did not confirm the association between total score and exclusive breastfeeding at discharge when adjusting for possible confounders. The limited number of fathers included in the study, together with the strong effect of parity and mode of delivery on exclusive breastfeeding rates at discharge, may be responsible for this result. The statistical power of our questionnaire in predicting exclusive breastfeeding at discharge was understandably limited, since it seems unrealistic to expect of any test to reliably predict such a complex outcome, bound for its very nature to be influenced by numerous factors. However, our results are in line with the current international literature [42] in highlighting how fathers more invested in breastfeeding and more informed about how it may influence their newborns’ feeding choices.

Therefore, there appears to be an ever-growing need for father-focused interventions to teach fathers how to better help and support their partners, thus expanding the classic mother-baby dyad to include them as well, as part of the breastfeeding team [29, 43].

We acknowledge that the present study has some limitations. Firstly, data were collected from a single Italian center; thus, our results and subsequent considerations may not apply to different settings. Specifically, the unique demographic of fathers participating in this study does not allow comparisons with other studies addressing the same topic in different populations. Furthermore, our study population consists of fathers who purposefully chose with their partner to deliver their baby in a BFHI-compliant hospital. This may have potentially determined a selection bias of fathers more sensitive and open to breastfeeding. Secondly, breastfeeding rates were evaluated only at discharge. A long-term follow-up would probably add more interesting information. Moreover, we acknowledge that all items of the questionnaire used are worded in the direction that favors breastfeeding, and, as such, may have led the subjects, resulting in higher scores than an instrument including questions worded more neutrally. Finally, it would have been interesting to compare knowledge and attitude toward breastfeeding between fathers whose partners aimed to breastfeed and fathers whose partners did not. Likewise, it would have been interesting to evaluate mothers’ knowledge and attitude toward breastfeeding together with their partners to understand if there is any interaction between the two. These topics could be addressed in future research.

However, our study provides valuable insight into the personal breastfeeding experience of fathers of newborns born at our center, since it addressed a relatively large number of fathers, whose answers were blinded to mothers, thus not influenced by their partners’ opinions.

## Conclusions

Socio-cultural changes are progressively pushing toward a greater involvement of fathers in what was once thought as a “women’s job” only. Within the multifaceted network of social support (family, friends, healthcare professionals) that revolves around mothers, fathers are especially influential in improving breastfeeding outcomes [42]. Providing fathers with more breastfeeding information both pre- and post-natally and prompting a favorable attitude toward it could improve long-term exclusive breastfeeding rates, although further studies, maybe multicentric and with a long follow-up period, are needed to confirm this hypothesis.

## Supplementary information

ESM 1 (DOCX 33 kb)

## Data Availability

Access to the dataset generated and analyzed during the current study is restricted to protect patient confidentiality and participant privacy. The dataset is available from the corresponding author upon reasonable request.

## Abbreviations

BFCI: Baby-Friendly Community Initiative
BFHI: Baby-Friendly Hospital Initiative
IBCLC: International Board Certified Lactation Consultant
NICU: Neonatal Intensive Care Unit
UNICEF: United Nations Children’s Fund
WHO: World Health Organization
