# Supplementary material for: From dyad to triad: a survey on fathers’ knowledge and attitudes toward breastfeeding
Source: Eur J Pediatr. 2021 Mar 29;180(9):2861–9. doi: 10.1007/s00431-021-04034-x (PMC8346446; doi:10.1007/s00431-021-04034-x)
Supplement: Supplementary file 1 — (DOCX 33 kb) [file 431_2021_4034_MOESM1_ESM.docx]

**Supplementary Table 1**

| **Questions** | | **Please fill out the questionnaire by yourself** | | | | |
| --- | --- | --- | --- | --- | --- | --- |
|  |  | **Strongly**  **Agree** | **Agree** | **Neutral** | **Disagree** | **Strongly**  **Disagree** |
| **1** | **Antenatal care** |  | | | | |
|  | I received sufficient information on breastfeeding management |  |  |  |  |  |
|  | Breastfeeding offers health benefits for infants |  |  |  |  |  |
|  | Breastfeeding offers health benefits for mothers |  |  |  |  |  |
|  | Breastfeeding benefits society |  |  |  |  |  |
| **2** | **Perinatal care** |  | | | | |
|  | Skin-to-skin contact after birth is a valuable opportunity |  |  |  |  |  |
| **3** | **Breastfeeding support** |  |  |  |  |  |
|  | I feel personally involved in feeding my baby |  |  |  |  |  |
| **4** | **Rooming-in** |  | | | | |
|  | Rooming-in affects breastfeeding initiation |  |  |  |  |  |
| **5** | **Responsive feeding** |  |  |  |  |  |
|  | Breastfeeding on-demand is beneficial |  |  |  |  |  |
| **6** | **Use of pacifier** |  | | | | |
|  | Breastfed infants should not use pacifiers in the first month of life |  |  |  |  |  |
| **7** | **Staff competency and discharge** |  | | | | |
|  | Information received during hospital stay and at discharge was clear |  |  |  |  |  |
| **8** | **Breastfeeding does not complicate everyday life** |  |  |  |  |  |
| **9** | **Mothers can breastfeed wherever they are** |  |  |  |  |  |

This table shows the questionnaire handed out to fathers enrolled in the study.

**Supplementary Table 2**

|  |  | **Total Score** |  |
| --- | --- | --- | --- |
|  |  | **Median [IQR]** | **p** |
| **Nationality** | **Italian** | 50 [46-52] | 0,293 |
|  | **Foreign** | 51 [49-53] |  |
| **Level of education** | **Secondary school** | 52 [48-53] | 0,584 |
|  | **High school** | 50 [46-53] |  |
|  | **University degree** | 50 [46-52] |  |
| **Age** | **≤ 36 years** | 50 [47-52] | 0,903 |
|  | **>36 years** | 49 [46-53] |  |
| **Parity** | **Primiparous** | 50 [46-52] | 0,962 |
|  | **Multiparous** | 49 [46-53] |  |
| **Previous feeding experience** | **None** | 50 [46-52] | 0,096 |
|  | **Exclusive BF** | 50 [47-53] |  |
|  | **Mixed feeding** | 49 [47-54] |  |
|  | **Bottle feeding** | 49 [42-49] |  |
| *Abbreviations: BF, breastfeeding.* | |  |  |

This table shows comparison of median total score values between subgroups.
